# A Meta-Analysis of Site-Specific Effects of Cathodal Transcranial Direct Current Stimulation on Sensory Perception and Pain

**DOI:** 10.1371/journal.pone.0123873

**Published:** 2015-05-15

**Authors:** Bita Vaseghi, Maryam Zoghi, Shapour Jaberzadeh

**Affiliations:** 1 Department of Physiotherapy, School of Primary Health Care, Faculty of Medicine, Nursing and Health Sciences, Monash University, Melbourne, Australia; 2 Department of Medicine, Royal Melbourne Hospital, The University of Melbourne, Melbourne, Australia; University of Bologna, ITALY

## Abstract

The primary aim of our meta-analysis was to evaluate the effects of cathodal transcranial direct current stimulation (c-tDCS) on sensory and pain thresholds (STh and PTh) in healthy individuals and pain level (PL) in patients with chronic pain. Electronic databases were searched for c-tDCS studies. Methodological quality was evaluated using the PEDro and Downs and Black (D&B) assessment tools. C-tDCS of the primary motor cortex (S1) increases both STh (P<0.001, effect size of 26.84%) and PTh (P<0.001, effect size of 11.62%). In addition, c-tDCS over M1 led to STh increase (P<0.005, effect size of 30.44%). Likewise, PL decreased significantly in the patient group following application of c-tDCS. The small number of studies precluded subgroup analysis. Nevertheless, meta-analysis showed that in all groups (except c-tDCS of S1) active c-tDCS and sham stimulation produced significant differences in STh/PTh in healthy and PL in patient group. This review provides evidence for the site-specific effectiveness of c-tDCS in increasing STh/PTh in healthy individuals and decreasing PL in patients with chronic pain. However, due to small sample sizes in the included studies, our results should be interpreted with caution. Given that the level of blinding was not considered in the inclusion criteria, the results of the current study should be interpreted with caution.

## Introduction

Cathodal transcranial direct current stimulation (c-tDCS) is one of the non-invasive brain stimulation techniques which depends on the parameters of the applied current, may induce decreased or increased corticospinal excitability [1, 2]. The inhibitory effect of c-tDCS has been recently utilized for treatment of different clinical conditions including pain management [3–5]. To understand how c-tDCS modulates pain, it should be noted that a large distributed network of brain sites are activated during pain processing [6] which collectively is called pain neuromatrix [7, 8]. Some parts of the pain neuromatrix are superficial, including the primary sensory cortex (S1), primary motor cortex (M1), and dorsolateral prefrontal cortex (DLPFC). Other areas of the pain neuromatrix such as the thalamus, insula, and anterior cingulate cortex, and pre-acuectal grey matter are deeper structures [9, 10].

Since S1, M1, and DLPFC make contributions to pain processing [11, 12], the site of stimulation should have a differential effect on pain relief. Some imaging studies have indicated that S1 is responsible for sensory discriminative component of pain, including stimulus localization, intensity and discrimination of pain quality [7, 10, 13–15]. Furthermore, functional connectivities between M1, ventro-lateral, and anterior thalamic nuclei affect medial thalamus, anterior cingulate cortex, and upper brainstem functions [16–18]. These connectivities are the means by which the central nervous system regulates the musculoskeletal system during painful conditions. Regardless of pain location, DLPFC affects cognition, attention, anticipation, and emotion aspects of pain during the pain processing [8, 15, 19–22]. There is also evidence that prefrontal cortex and the anterior cingulate cortex are activated during pain expectation [23] and pain-induced anxiety [24].

Pain can be operationalized into key variables including sensory threshold (STh) and pain threshold (PTh) in healthy individuals [15, 25, 26] and pain level (PL) in patients with chronic pain [27, 28]. Recent investigations have demonstrated that c-tDCS of superficial areas of pain neuromatrix induces excitability decrease [1] which results in STh/PTh increase [29, 30] and PL decrease [27]. In contrast, others report no effect on these behavioral variables [31]. These results raise a very important question: what is the evidence for the effectiveness of c-tDCS in modulating pain according to the site of stimulation? To date, no meta-analysis has drawn together the abundant evidence from the existing literature on the effects of c-tDCS over superficial areas of pain neuromatrix on STh/PTh and PL to reach a firm conclusion about the efficacy of c-tDCS in pain management. In the current study, we aimed to investigate the site-specific effects of c-tDCS on STh/PTh in healthy individuals and PL in patients with chronic pain.

## Methodology

### Inclusion criteria

English-language articles describing all types of study designs, including parallel or cross over studies, were included in the current study regardless of blinding. Studies that utilized c-tDCS on the S1, M1, or DLPFC in healthy individuals or patients experiencing chronic pain were recruited if the participants were over 18 years of age and either healthy or had experienced chronic pain for more than three months [6, 32], the outcome measures of interest were the visual analogue scale (VAS) in the patient group or STh/PTh in the healthy group, and sham tDCS or active control was applied (Table 1).

**Table 1:** Inclusion and exclusion criteria for identified studies.

	Inclusion	Exclusion
**Participants**	- Studies in which individuals were over 18 years of age	- Studies involving individuals suffering from other type of diseases (i.e., depression or other type of mental disorders, cancerous pain
	- Either healthy or suffering from chronic pain (no limits were applied to the type (musculoskeletal, neural, or central pain syndrome), anatomical location	- Studies on patients with primary symptoms other than pain (i.e., depression or schizophrenia)
**Intervention**	- Studies that involve c-tDCS and Sham as intervention of interest	
**Comparison**	- Studies in which the comparison of interest is “no treatment”/sham treatment	- Other control group
	- Before and after c-tDCS	
**Outcomes**	- Studies in which the outcome measure of interest were Numeric Analogue Scale (NAS) measured by Quantitative Sensory Testing (QST) method and LEP amplitude in healthy individuals and VAS in patients with chronic pain	- Other type of evaluation of sensory perception, pain threshold, and PL (Measured by rTMS, fMRI, PET, and Paired TMS)
**Trial design**	- Randomized control trial, controlled clinical trials, and pre-post trials	- Review articles
		- Selective review
**Data reported**	- Data that enable analysis and estimation of the effects of c-tDCS and Sham on STh, PTh, and PL must be reported	
**Type of publications**	- Published in a peer-reviewed journal, regardless of the year of publication	Non English articles
	- As the services for translation do not exist, only English publications will be considered	

Given the fact that M1, S1, and DLPFC are the only superficial areas of pain neuromatrix which are accessible for stimulation by tDCS, we included studies investigated the effects of c-tDCS on these areas in both healthy subjects (Table 2) and patient groups with chronic pain regardless of their pathology (Table 3). All modalities that evoked a sensory or painful sensation were included (i.e., laser, heat, cold and mechanical stimuli). Chronic pain was specified as a refractory pain which is resistant to medical intervention or drug management for more than three months [6, 32]. We included studies that placed electrodes over M1, S1 or DLPFC regions.

**Table 2:** Study characteristics and outcome measure in healthy individuals.

Included Studies	Trial design	No. Participants	Stimulation method	Outcome measure	Intervention	Stimulated area
**Antal et al. 2008**	Pre-Post test	10	LASER	NAS, LEP	a-tDCS, c-tDCS	S1
**Bachmann et al. 2010**	Single blinded, Crossover trail	8	QST	NAS	a-tDCS, c-tDCS	M1
**Boggio et al. 2008**	Double blinded, sham controlled	20	ES	NAS	a-tDCS	V1^1^, M1^2^, DLPFC^3^
**Csfcsak et al. 2009**	Pre-Post test	10	LASER	NAS, LEP	a-tDCS, c-tDCS	M1
**Grundmann et al. 2010**	Pre-Post test	12	QST	NAS	a-tDCS, c-tDCS	S1^6^
**Hansen et al. 2010**	Pre-Post test	19	ES	NAS, PREP^4^, BR^5^	a-tDCS, c-tDCS	M1
**Rogalewski et al. 2004**	Single blinded, Sham controlled	13	Tactile perception	NAS	a-tDCS, c-tDCS	S1
**Terney et al. 2008**	Single blinded crossover trial	15	LASER	NAS, LEP	a-tDCS, c-tDCS	M1

1. Primary visual cortex

2. Primary motor cortex

3. Dorsolateral prefrontal cortex

4. Pain Related Evoked Potential

5. Blink Reflex

6. Somatosensory cortex

**Table 3:** Study characteristics and outcome measure in patients with chronic pain.

Included Studies	Trial design	No. Participants	Patients	Stimulation area	Intervention	Outcome measure
**Antal et al. 2011 a**	Double blinded sham control	26	Chronic Migraine	V14	c-tDCS	VAS
**Mendonca et al. 2011**	Double blinded randomised control	30	Fibromyalgia	M1	a-tDCS, c-tDCS	VNS^1^

1. Visual Numeric Scale

### Exclusion Criteria

Studies were excluded if they did not involve brain stimulation, the duration of symptoms for patient groups was not clear, the study used deep brain stimulation, transcranial magnetic stimulation, repetitive transcranial magnetic stimulation, or electrical stimulation with pulse currents (Table 1). In addition, studies that used a-tDCS or indirect forms of stimulation (caloric vestibular stimulation or occipital nerve stimulation) were excluded.

### Outcome measures

The outcome measures for STh and PTh were percentage changes in stimulus intensities at which participants reported the onset of sensation (STh) or onset of pain (PTh). For PL in the patient group, we pooled studies that used VAS (Tables 2 and 3).

Because the included trials involved post-intervention assessments at varying time points, they were partitioned into short-term and long-term outcomes. “Short-term” was arbitrarily defined as less than one hour after intervention. If a trial had multiple assessments during that period, the assessment performed closest to the intervention was used. “Long-term” was defined as greater than one hour after intervention; long-term outcomes were not included in the current meta-analyses.

### Methods for identifying studies

To locate eligible articles, a broad search was performed on all English literatures through relevant databases including PubMed, Physiotherapy Evidence Databases (PEDro), CINAHL, Cochrane Central Register of Controlled Trials, Scopus, PROQuest, SPorTDiscuss, Australian Medical Index, Ovid Medline, EBM Review, Cochrane, Meditex and PsycINFO from their inception to December 2014. All reference lists of retrieved papers were searched to identify additional relevant articles missed in the initial search strategy. The key words were “transcranial direct current stimulation”, “tDCS”, “sensory perception”, “pain”, “pain perception”, “pain tolerance”, “sensory threshold”, “pain threshold”, “sensory stimulation” and “pain trigger”.

### Selection of the included studies

Considering the inclusion criteria, both randomized and non-randomized trials were selected. Two independent raters (BV and SJ) reviewed the title and abstract of all selected papers. If the information in the title and abstract was insufficient to make a decision, the reviewers assessed the full paper to include or exclude the study. All included studies were then double-checked by a full-text appraisal. If the reviewers disagreed, resolution was attempted by discussion. If resolution was not achieved, the third reviewer (MZ) was consulted.

### Risk of bias assessment

**Fig 1:**
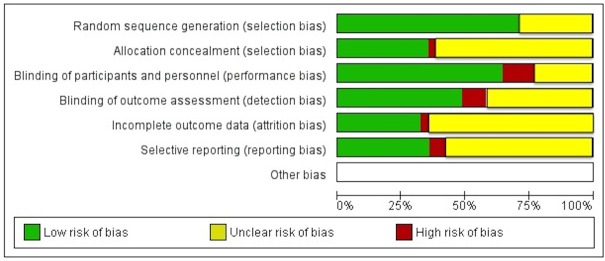
Risk of bias graph: Review authors’ judgments about each risk of bias item presented as percentages across all 10 included studies.

The risk of bias and methodological quality of included studies were evaluated by the assessed method defined in Chapter 8 of the *Cochrane Handbook for Systematic Reviews of Interventions Version 5*.*1*.*2* [33]. Fig 1 is a methodological quality graph for all included studies.

PEDro scale was used for further quality assessment [34, 35] in which there are 10 criteria for internal validity; studies are awarded a point for each criterion met. The PEDro cut-points are 9–10, excellent; 6–8, good; 4–5, fair and below 4, poor [36]. For non-randomized controlled trials, Down and Black tool (D&B) was used [37] (Table 4).

**Table 4:** Quality assessment of included studies.

	Included studies	PEDro (1999)	D & B (Downs and Black, 1998)
**Healthy group**	Bachmann et al. 2010	7	-
	Rogalewwski et al. 2004	7	-
	Terney et al. 2008	-	16
	Csfcsak et al. 2009	-	17
	Grundmann et al. 2010	-	16
	Henssen et al. 2010	-	17
	Antal et al. 2008	-	18
	Boggio et al. 2008	-	18
**Patient group**	Mendonca et al. 2011	7	-
	Antal et al. 2011	8	-

### Outcome measures

Our primary outcome measures were the STh and PTh of healthy individuals and PL in patients who suffered from chronic pain. STh is usually measured by quantitative sensory testing using mechanical, vibration or thermal methods [38]. The STh is defined as the level of stimulus intensity at which sensation was detected for the first time. PTh is defined as the level of stimulus intensity at which pain is detected. PL in patients with chronic pain was defined as the average pain that they experience during a day, usually measured by the VAS [39].

### Subgroup analysis and intervention of heterogeneity

The heterogeneity of included studies was evaluated by Chi^2^ test and I^2^ statistic. There are two subgroups in each meta-analysis assessing the effects of c-tDCS on STh and PTh in healthy individuals: (i) c-tDCS of S1, and (ii) c-tDCS of M1. Due to the limited included studies in patient group, the overall effect of c-tDCS on PL with no subgroup analysis was assessed in the patient group.

### Data extraction

The following data were extracted from the included studies: study design, characteristics of subjects, outcome measures; stimulated areas in healthy (Table 2) and patient group (Table 3). C-tDCS parameters, the position and size of active electrodes are also summarized in Table 5. We asked the corresponding author(s) to send us the mean ± SD of desired outcome measures. Where the requested data were not provided, mean ± SD values were extracted from tables or pooled from graphs using Plot Digitizer software [40]. In studies that did not report standard deviation (SD), we used the formula SD = SE√n (n = number of subjects in each group) [33].

**Table 5:** c-tDCS parameters in healthy individuals.

Included studies	Electrode size (cm^2^)	Intensity (mA)	Current density (mA/cm^2^)	Time (min)	Electrode Position
**A: Healthy group**					
**Antal et al. 2008**	35	1	0.029	15	2cm posterior to ADM^1^ hot spot
**Bachmann et al. 2010**	35	1	0.029	15	C3
**Boggio et al. 2008**	35	2	0.057	5	C3, F3, Oz
**Csfcsak et al. 2009**	35	1	0.029	10	C3
**Grundmann et al. 2010**	35	1	0.029	15	C3
**Hansen et al. 2010**	16	1	0.063	20	Cz, 1 cm above the supraorbital nerve
**Rogalewski et al. 2004**	35	1	0.029	7	C4
**Terney et al. 2008**	35	1	0.029	15	ADM^1^ hot spot
**B: Patient group**					
**Antal et al. 2011 a**					
**Mendonca et al. 2011**	16	1	0.063	20	C3
	16, 80	2	0.125, 0.0125	20	C3

1. Abductor digiti minimi

Plot Digitizer, a java program, was used to digitize data point off of scanned plots [33] was used to digitize scanned plots of functional data. Statistical significance of the difference between extracted data was calculated with 95% of confidence intervals (CIs) by RevMan software, version 5.1 (Cochrane Collaboration, 2008) [41]. RevMan is adjusted to calculate small sample bias [33]. Extracted data were entered into the meta-analysis using the generic inverse variance method as suggested in the *Cochrane Handbook for Systematic Reviews of Interventions* [33].We used a random effects model to conduct separate meta-analyses for different forms of stimulation (c-tDCS and sham). Where more than one data point was available for short term outcomes, we used the first post stimulation measure. Two forest plots were generated for each outcome measure. In the first one, the percentage changes in STh, PTh and PL after applying c-tDCS compared to baseline values were assessed. In the second one, the percentage changes in STh, PTh and PL after c-tDCS were compared to the percentage changes after effects of sham stimulation (Table 5). Based on the *Cochrane Handbook for Systematic Reviews of Interventions*: “Standard mean difference (SDM) is used to measure effect size when the trials all assess same outcomes, but measured in a variety of ways. As a result, the effect measure for STh/ PTh in healthy and PL in patient groups was assessed by SMD by which we had this opportunity to clarify the degree of improvement or no improvement in our outcome measures after the intervention. The SMD calculation in RevMan software is given by:

$$SMD\;=\;\frac{Difference\;in\;mean\;outcome\;between\;groups}{Standard\;deviation\;of\;outcome\;among\;participants}$$

## Results

### Identification and selection of studies

**Fig 2:**
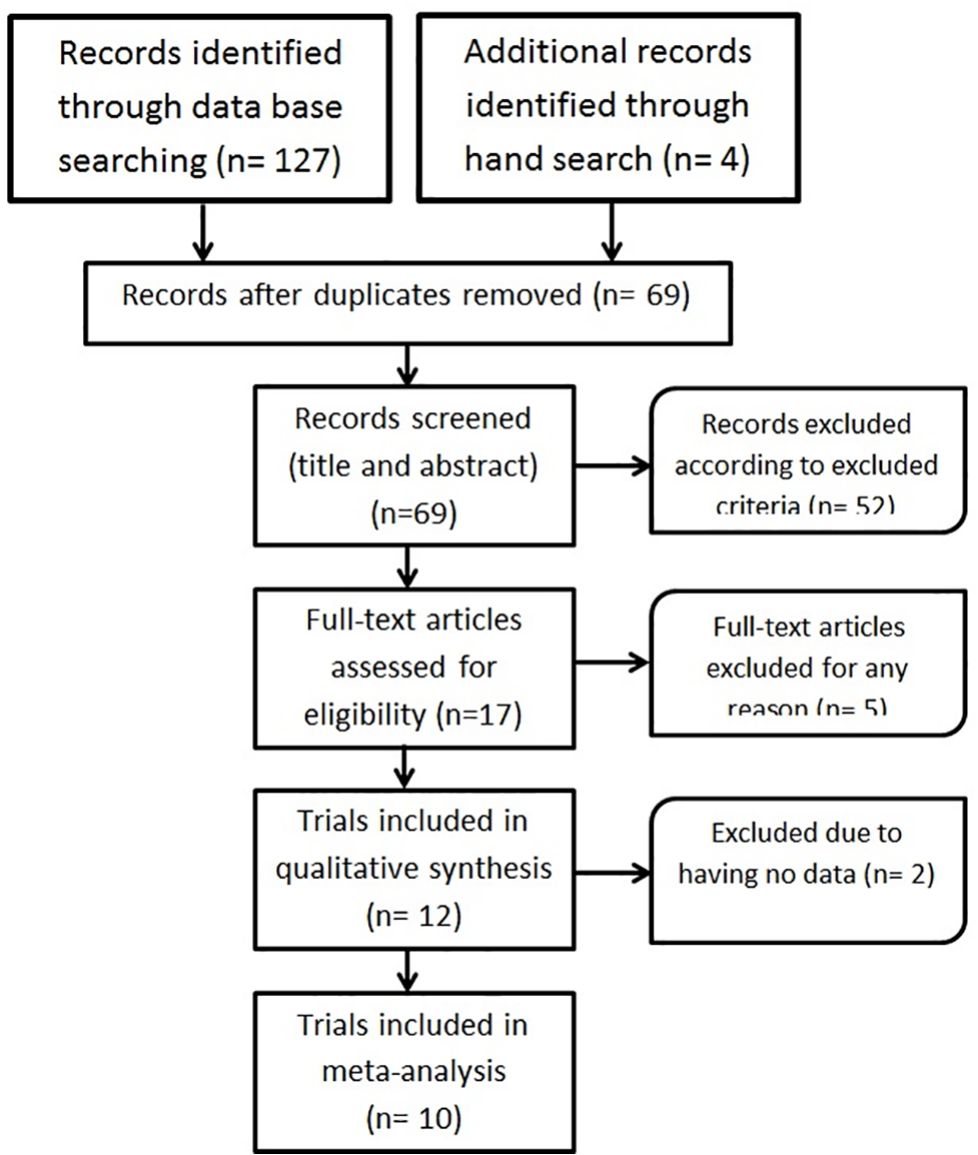
Flowchart study selection.

The search strategy identified 131 studies, including 62 duplicates. Screening by title and abstract, 17 studies, including 12 studies in healthy and 5 in patient group, were eligible to review in which three studies in the healthy and one study in the patient group were identified from manual searching the reference lists of included studies. Five studies, which did not meet inclusion criteria, were excluded. As requested data were not provided from corresponding authors and graphs or tables of two papers, they were excluded. Therefore, the final number of study is 10 (8 in healthy and 2 in patient group) (Fig 2).

### Risk of bias and quality assessment

No study was judged to have a low risk of bias across all criteria. Fig 1 summarizes the risk of bias assessment results. All trials had unclear or inadequate bias control in one or more of the domains for the assessment of risk of bias. Lack of blinding of participants and personnel was the major potential source of bias in the current meta-analysis. Also, allocation concealment and completeness of outcome data were unclear in more than 50% of studies, representing a moderate risk of bias. However, the PEDro score was 7 in the healthy group (mean score of 7/11) and ranged between 7 and 8 in the patient group (mean score of 7.5/11), which is in the range of good quality controlled trials. Mean score of 12/27 in D&B quality checklist indicates that the mean quality checklist is medium in the healthy group. Table 3 shows the PEDro and D&B scores of the studies.

### Participants in included studies

In total across the included studies, 107 healthy individuals and 56 patients with chronic pain received c-tDCS and sham for VAS measurement. All studies assessed the effect of c-tDCS in one or more of the M1, S1, or DLPFC. In patients with chronic pain, the average VAS score was more than 5.

No study in the healthy group involved stimulating the DLPFC to measure STh/PTh. Furthermore, no study could be identified on the effects of S1 c-tDCS on PL. Due to the patient group containing only two studies, it was impossible to evaluate the site-specific effects of c-tDCS in this group.

### Pooled data analysis

For all studies the standard error (SE) was calculated from the 95% confidence interval of the standardized mean difference and entered into the meta-analysis using the generic inverse variance method. Pre-post c-tDCS studies and active/sham studies were evaluated to assess whether c-tDCS can change STh, PTh, and PL, and whether studies using a sham group as a control produced results significantly different from those of pre-post studies. The percentage changes before and after applying c-tDCS and sham were calculated and pooled in meta-analysis.

### Effects of c-tDCS on STh in healthy participants

**Fig 3:**
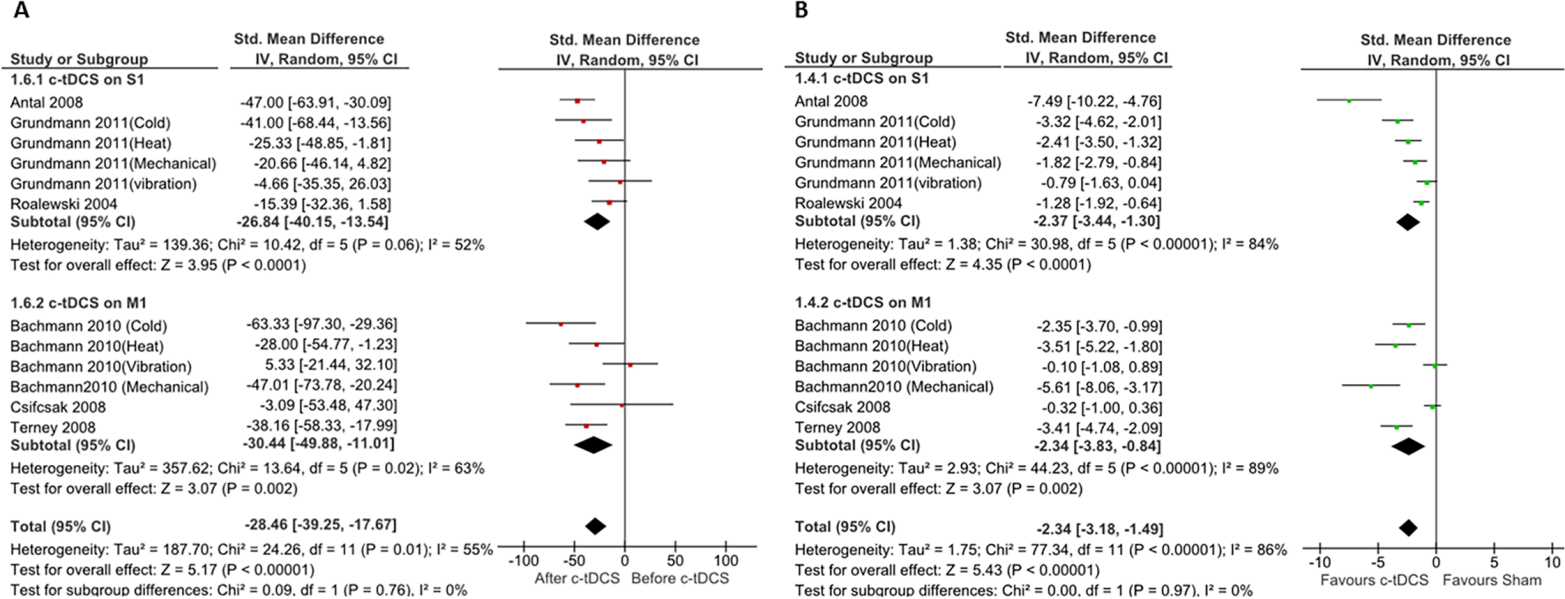
Forest plots of sensory threshold changes in healthy individuals. mparison of percentages of sensory threshold changes before and after c-tDCS (A), and comparison of after effects of sensory threshold changes between active and sham c-tDCS (B). Subgroup analysis: studies of M1 and S1 stimulation. ■ = the effect size for one trial; horizontal line = 95% of confidence interval; ◆ = pooled effect size for all trials. CI: confidence interval, IV: inverse variance.

Fig 3A summarizes the pooled data (percentages of changes) extracted from six studies on healthy individuals [29–31, 42–44]. The pooled analysis of three studies (n = 81) in S1 subgroup and three studies (n = 61) in M1 subgroup showed that c-tDCS of both S1 and M1 increased STh significantly. The heterogeneity of S1 stimulation was (I^2^ = 84%) and the percentages of STh increase was 26.84% (P< 0.0001, 95% CI, (from 40.15% to 13.54%)). The heterogeneity for M1 stimulation was (I^2^ = 69%), and the results suggest a significant benefit of c-tDCS on M1 ((28.25%, (95% CI 53.49%, 3.01%), P = 0.02)).

Overall, our meta-analysis of pooled data from all included studies indicates significant STh increase (P<0.00001) in healthy individuals with a main effect size of 27.30% (95% CI, (from 39.19 to 15.42%) (Fig 3A).

Fig 3B shows the result of comparison of sham and active c-tDCS. The results of meta-analysis of pooled studies demonstrated that there are significant STh changes in both S1 (pooled SMD: -2.37, (95% CI, (from -3.44 to -1.30)), P < 0.0001) and M1 (pooled SMD: -2.34, (95% CI, (from -3.34 to -1.49)), P = 0.002) subgroups. Forest plot and meta-analysis results also indicated a significant difference between sham and active c-tDCS (P<0.00001).

### Effects of c-tDCS on PTh in healthy participants

**Fig 4:**
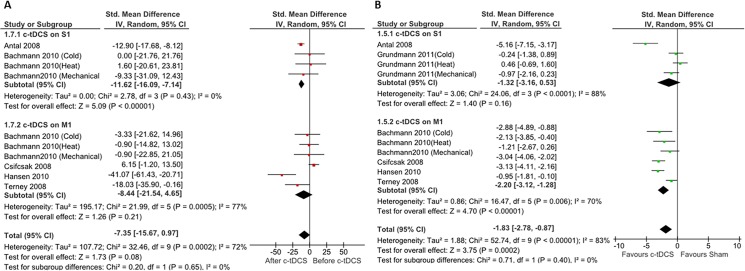
Forest plots of pain threshold changes in healthy individuals. Comparison of percentages of pain threshold changes before and after c-tDCS (A), and comparison of after effects of pain threshold changes between active and sham c-tDCS (B). Subgroup analysis: studies of M1 and S1 stimulation. ■ = the effect size for one trial; horizontal line = 95% of confidence interval; ◆ = pooled effect size for all trials. CI: confidence interval, IV: inverse variance.

Six studies assessed the effect of c-tDCS on PTh in healthy individuals [29, 31, 44–46]. Two studies (n = 46) stimulated S1 and four (n = 72) focused on M1 stimulation. The subgroup results demonstrated c-tDCS generated a significant PTh increase in the S1 subgroup, with a mean effect size of (11.62%, (95% CI, from 16.09% to 7.14%), P<0.00001) and heterogeneity of 0%; this was not the case in the M1 subgroup and there was no significant change in PTh after applying c-tDCS on M1 (Fig 4A). The analysis also indicated no significant overall effect on PTh (P = 0.08).

As can be seen in Fig 4B, meta-analysis showed that while there is a significant difference between sham and active c-tDCS of M1 (pooled SMD: -2.20, (95% CI, (from -3.12 to -1.28)), P < 0.00001), there is no significant difference between sham and active c-tDCS of S1 (pooled SMD: -1.32, (95% CI, (from -3.16 to 0.53), P = 0.16).

### The effect of c-tDCS on PL in patients with chronic pain

**Fig 5:**
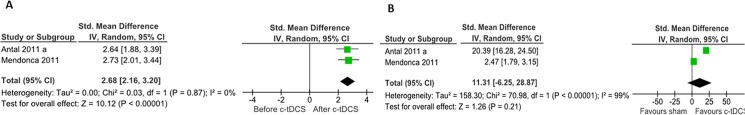
Forest plots of pain level changes in patients with chronic pain. Comparison of percentages of pain level changes before and after c-tDCS (A), and comparison of after effects of pain level changes between active and sham c-tDCS (B). Subgroup analysis: studies of M1 and dorsolateral prefrontal cortex (DLPFC) stimulation. ■ = the effect size for one trial; horizontal line = 95% of confidence interval; ◆ = pooled effect size for all trials. CI: confidence interval, IV: inverse variance.

We had insufficient data to investigate the effect of site of stimulation in the patient group, but we could investigate the overall effect of c-tDCS on PL in patients with chronic pain. Data were available from two studies (n = 56) [27, 28]. Regarding heterogeneity of included studies (I^2^ = 0%), the evidence suggested that applying c-tDCS resulted in a significant decrease in PL in patients with chronic pain. The pooled effect was 2.68 (95% CI, (from 2.16 to 3.20)), P < 0.00001) (Fig 5A).

The comparison of active and sham c-tDCS indicates that a non-significant difference in PL after applying active and sham tDCS (pooled SMD: 11.31, (95% CI, (from -6.25 to 28.87)), P = 0.21) (Fig 5B).

### The impact of individual studies on the overall results

The effect of each included study on the pooled effect size of overall analyses was examined in both healthy and patient groups.

### C-tDCS and STh/PTh in healthy individuals

**Fig 6:**
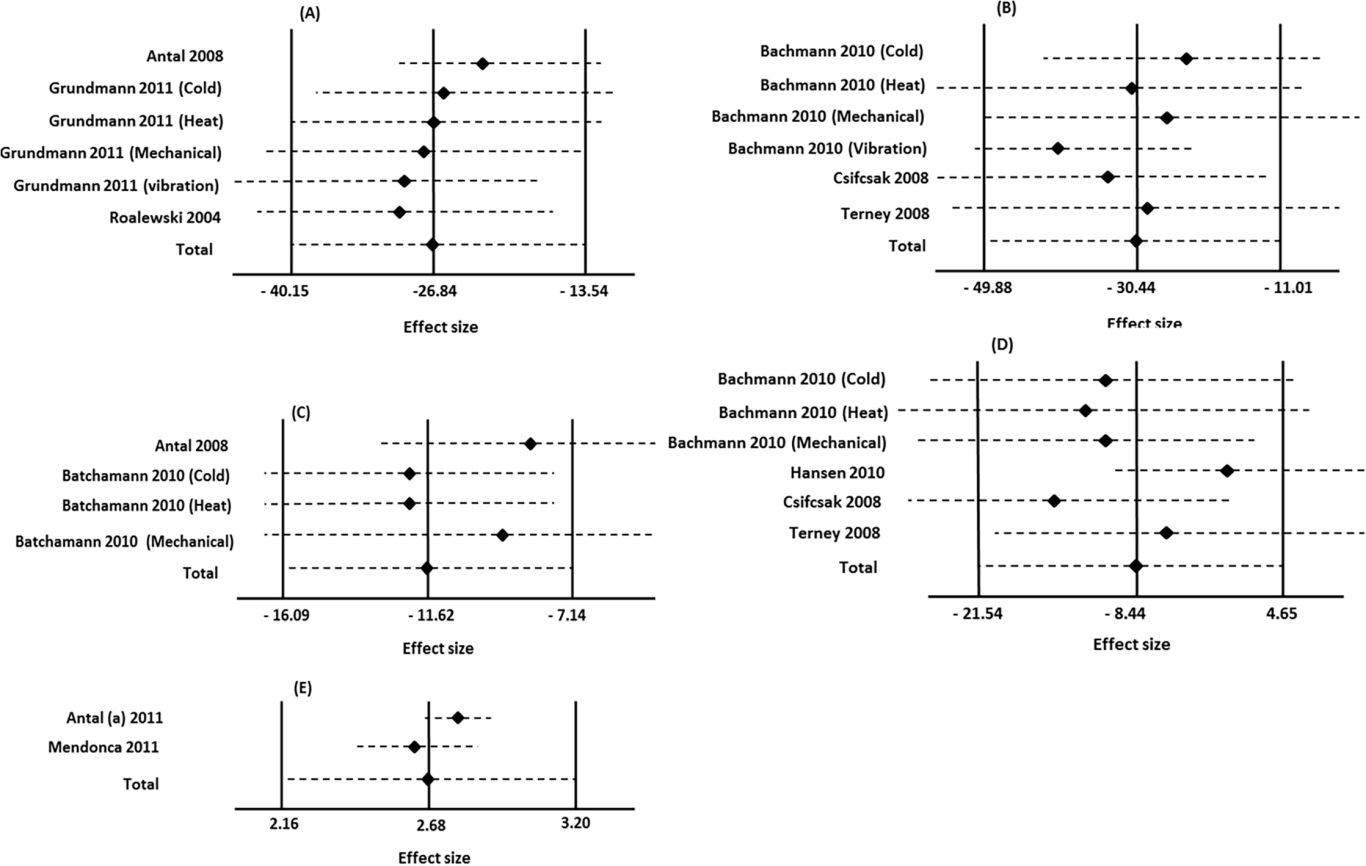
Assessment of the individual influence of included studies evaluating the after effects of c-tDCS on outcome measures. The Impact of single studies on overall effect size in studies evaluating the effect of c-tDCS of S1 (A) and M1 (B) on sensory threshold, c-tDCS of S1 (C) and M1 (D) on pain threshold in healthy individuals, and pain level (E) in patients with chronic pain were evaluated. The effect sizes are Cohen’s d (SMD) and error bars represent the 95% confidence interval. The left, middle, and right vertical lines are indicator for the minimum, mean, and maximum value of total effect size respectively.

Based on the result of sensitivity analysis, the total effect size of meta-analysis evaluating after effects of S1 c-tDCS on STh did not change if any one single study was excluded although the pooled data was slightly decreased by excluding the study of Antal et al. 2008 (P = 0.001) and Grundmann et al. 2011 (cold and heat: P = 0.001, and mechanical: P = 0.0005). Exclusion of Grundmann (vibration) and Roalewski et al. (2004) data had no effect on the overall effect size (Fig 6A). The effect size of STh after c-tDCS of M1 was slightly decreased when Terney et al. (2008) (P = 0.03) and Bachmann et al. (2011) studies (cold and heat: P = 0.01, and mechanical: P = 0.02) were excluded. Conversely, the pooled dada increased after excluding that part of Bachmann’s study evaluating the effect of M1 c-TDCS on vibration (Fig 6B).

The sensitivity analysis showed that excluding Antal et al. (2008) study and Bachmann et al. (2010) (mechanical) investigating the after effects of S1 c-tDCS on PTh in healthy individuals decreased the overall effect size to 0.68 and 0.05 respectively. Excluding other studies had no effect on pooled effect (Fig 6C). In addition, the pooled data did not changed by excluding each study evaluating the M1 c-tDCS on PTh (Fig 6D).

**Fig 7:**
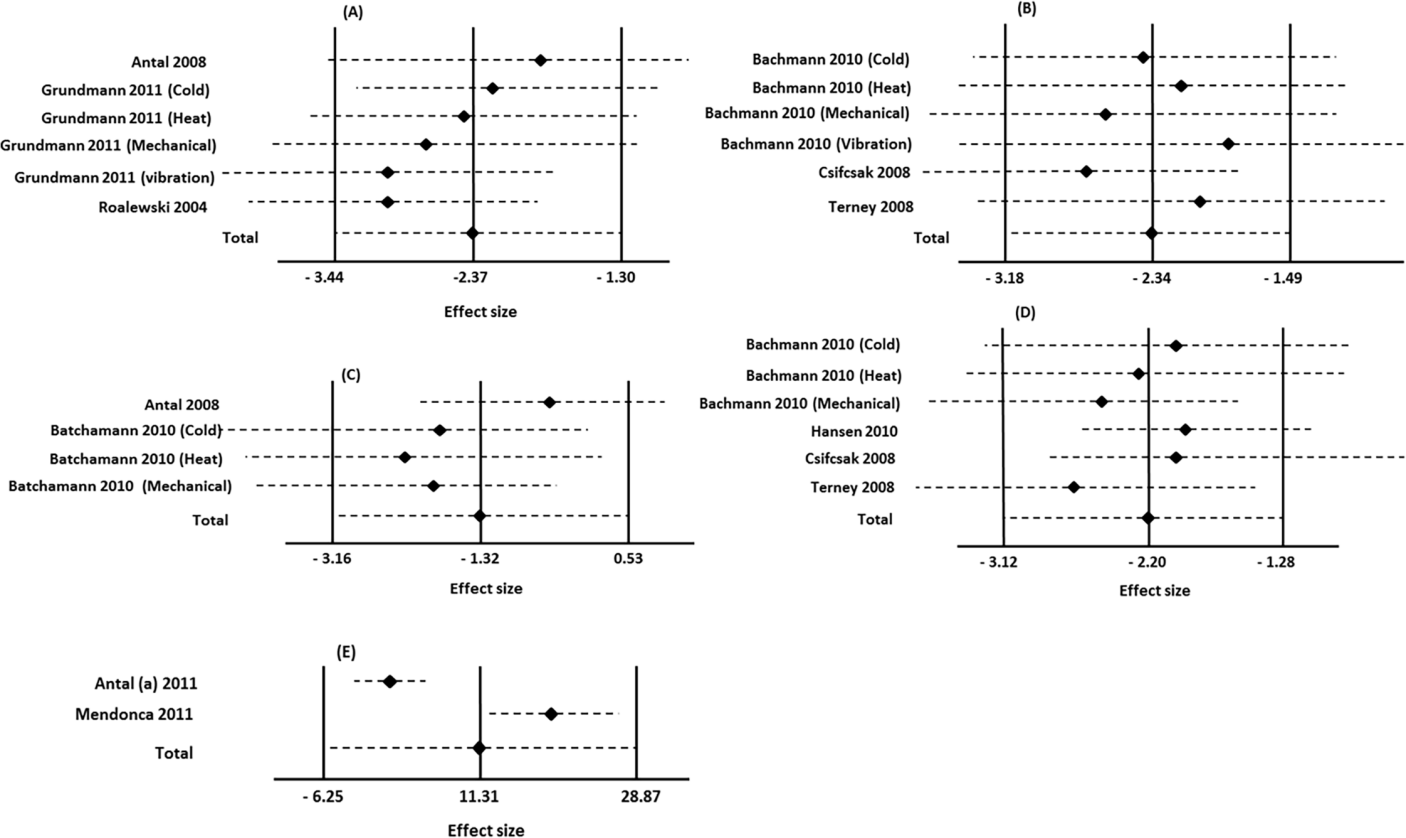
Assessment of the individual influence of included studies evaluating the effect of active and sham c-tDCS on outcome measures. The Impact of single studies on overall effect size in studies evaluating the effect of active and sham c-tDCS of S1 (A) and M1 (B) on sensory threshold, c-tDCS of S1 (C) and M1 (D) on pain threshold in healthy individuals, and pain level (E) in patients with chronic pain were evaluated. The effect sizes are Cohen’s d (SMD) and error bars represent the 95% confidence interval. The left, middle, and right vertical lines are indicator for the minimum, mean, and maximum value of total effect size respectively.

Likewise, the impact of individual studies on the meta-analyses comparing active and sham c-tDCS on STh and PTh were evaluated (Fig 7A–7D). For meta-analysis on STh, the pooled effect size would decrease to 0.0002 if the studies of Grundmann et al. (2011) on cold, heat, or mechanical sensation omitted. Exclusion of other including studies had no effect on the overall pooled effect (Fig 7A). The results also indicated that the overall effect size of meta-analysis compared the effects of M1 c-tDCS and sham on PTh did not change after exclusion of each single study. However, pooled effect size reached to 0.01 by omitting Bachmann et al. (2010) experiments (Fig 7B).

The result of sensitivity analysis focused on the effects of active and sham S1 (Fig 7C) and M1 (Fig 7D) c-tDCS on PTh in healthy individuals indicated that excluding each individual study had no effect on the overall pooled effect size.

### C-tDCS and PL in Patients with chronic pain

The result of sensitivity analysis in overall effect size in meta-analysis evaluating the after effects of c-tDCS on PL in patient group illustrated that omitting each study had no effect on overall effect size (Fig 6E). In contrast, in meta-analysis comparing sham and active c-tDCS on PL, exclusion of Antal et al. (2011a) study would increase the pooled effect size to 0.002 (Fig 7E).

## Discussion

Our meta-analysis involved eight studies of the effects of c-tDCS on STh and PTh in healthy individuals and two studies of the effects of c-tDCS on PL in patients with chronic pain. We aimed to evaluate the effectiveness of c-tDCS in increasing STh/PTh and PL according to the site of stimulation. The results of subgroup analyses in healthy individuals showed that, compared to baseline values, c-tDCS of S1 increased both STh and PTh in healthy individuals. Furthermore, c-tDCS of M1 led to significant STh but not PTh increase. Due to the scarcity of studies applying c-tDCS in patients with chronic pain, we could not conduct subgroup analysis in the patient group, but c-tDCS significantly decreased PL in the patient group overall. Similar results were found from comparison of active c-tDCS and sham stimulation, except for c-tDCS of S1, in which no significant difference was observed. More studies are needed to reach a firm conclusion in this regard.

### The effects of c-tDCS on STh in healthy individuals

In spite of the small number of studies available, the results of our meta-analysis showed that application of c-tDCS on both M1 and S1 increases STh in healthy individuals. Of six studies, which involved the S1, five reported c-tDCS of the S1 increased STh [30, 43, 45], and one study concluded that c-tDCS of S1 had no effect on STh [30] (Fig 3).

Four of six studies reported a significant increase in STh after c-tDCS of M1, and the remaining two failed to show such an increase. In addition, our comparison of the after effects of active c-tDCS and sham stimulation of the M1 and S1 subgroups demonstrated a significant increase in STh.

As can be seen in Fig 3, the heterogeneity of each subgroup and overall heterogeneity was moderate. We used sensitivity analysis [47] to assess the impact of excluding the studies with high risk of bias; the results demonstrate no changes in heterogeneity, which indicate insufficient data from which to draw a firm conclusion. Also, the different stimulation methods applied in the included studies to assess sensory threshold (laser, heat, cold, and electrical stimulation) could be another reason for the moderate heterogeneity.

Due to the fact that unmyelinated C-fibers transmit the sensation of warmth and small myelinated Aδ fibers transmit the sensation of non-painful cold [48], pooling data for different sensation (cold, warm, vibration, mechanical, etc.) might affect the results. The findings of Summers (2004) and Oliviviero (2005) suggested that repetitive transcranial magnetic stimulation of the somatosensory cortex increased cold perception but not warmth perception [49, 50]. Studies with larger sample sizes using the same methods of sensory threshold assessment will increase statistical power and decrease heterogeneity.

There are several basic mechanisms to explain the increased after effects of c-tDCS. First, prolonged constant electric field alters ionic concentration in stimulated area, which led to migration of transmembrane proteins and acid-base balance changes [51, 52]. Second, direct currents dissociate pure water to H^+^ and OH^-^ [53] resulted in acid-base balance changes by inducing acidosis or alkalosis that in turn strongly affect membrane, receptor and cell function [54]. Because changes in intracellular pH and (Ca ^2+^) are tightly correlated [55], one possibility is that c-tDCS changes pH and Ca ^2+^ concentration and increases STh

### The effects of c-tDCS on PTh in healthy individuals

Meta-analysis of the included studies demonstrates conflicting results in two subgroups. Compared with the baseline conditions, c-tDCS of S1 significantly increased PTh, whilst c-tDCS of M1 had no effect on PTh. Additionally, our comparison of the immediate after effects of active c-tDCS and sham stimulation of M1 demonstrated a significant difference in PTh. We also found no significant difference in PTh between sham stimulation and c-tDCS of S1.

The included studies used a wide range of stimulation parameters and methods, which might explain the substantial heterogeneity.

### The effect of c-tDCS on PL in patient group

The overall effect of c-tDCS was significant decreases in PL. Significant differences in PL of patients after application of c-tDCS and sham stimulation indicate the efficacy of c-tDCS in pain reduction. Due to the low number of c-tDCS studies in our patients group and its substantial heterogeneity, it was impossible to review site-specificity effects of c-tDCS on PL in different subgroups. The small number of included studies and participants, different pathology, site of stimulation, and stimulation parameters created substantial heterogeneity in the patient group data.

The results of our meta-analyses are in line with the conclusions from published systematic reviews [56–58], which allows us to conclude that c-tDCS can relieve pain in patients with chronic pain; however, more c-tDCS studies in chronic pain patients with different pathologies and sites of stimulation are recommended to improve the quality of the evidence.

The exact mechanisms underpinning the effects of c-tDCS on pain relief are not clear yet, but recent evidence categorized the effects of c-tDCS into two types: immediate after effects and long-lasting effects [59, 60]. The immediate after effects of c-tDCS can be explained by changes in the acid-base balance of neuron membranes [54, 61], excitability diminution [62], and consecutive reduction of NMDA receptor activity [62–64]. As a result, direct changes in membrane function, outside of synapses, change the activity of NMDA receptors indirectly and decrease the function of brain areas related to pain management [47, 59]. Based on the Kinkelin’s study (2000) it can be concluded that the after effects of tDCS do not arise from NMDA synaptic involvement alone. Although NMDA receptors are present on peripheral axons [65], they have not been reported on axons in the CNS [60, 63, 64].

### Quality of evidence

Assessor blinding status was not clearly reported which affect both homogeneity and pooled effect size. Regarding an epidemiological study, incomplete blinding in controlled trials may exaggerate the effect size by 25% [66]. Recently, O’Connell et al. (2012) reported that proper blinding is not possible in a study that uses a current intensity of 2 mA or greater. Though this conclusion was challenged by Russo et al. (2013) and Palm et al. (2013), the implication is that the overall quality of the evidence for the effects of c-tDCS on STh/PTh assessment in healthy individuals and PL assessment in patients with chronic pain is low and should be considered cautiously [67, 68]. Results should be replicated using a current intensity for which blinding is universally accepted as possible. Therefore, the effect size of our meta-analysis of STh and PTh measurements may be affected by incomplete blinding of included studies.

### Potential bias in the review process

Substantial variation exists between the included studies of c-tDCS in both the healthy and patient groups. Studies varied in terms of the stimulation parameters used, gender and range of ages of participants, and the number of treatment sessions, all of which increased the heterogeneity of subgroups. This heterogeneity was reflected in the I^2^ statistics for the overall c-tDCS meta-analyses. In addition, several studies in the healthy group used heat and others used cold stimuli for assessment of STh/PTh, and these different stimuli activate different pathways, so this can be considered as a source of methodological bias in final responses.

### Publication bias

**Fig 8:**
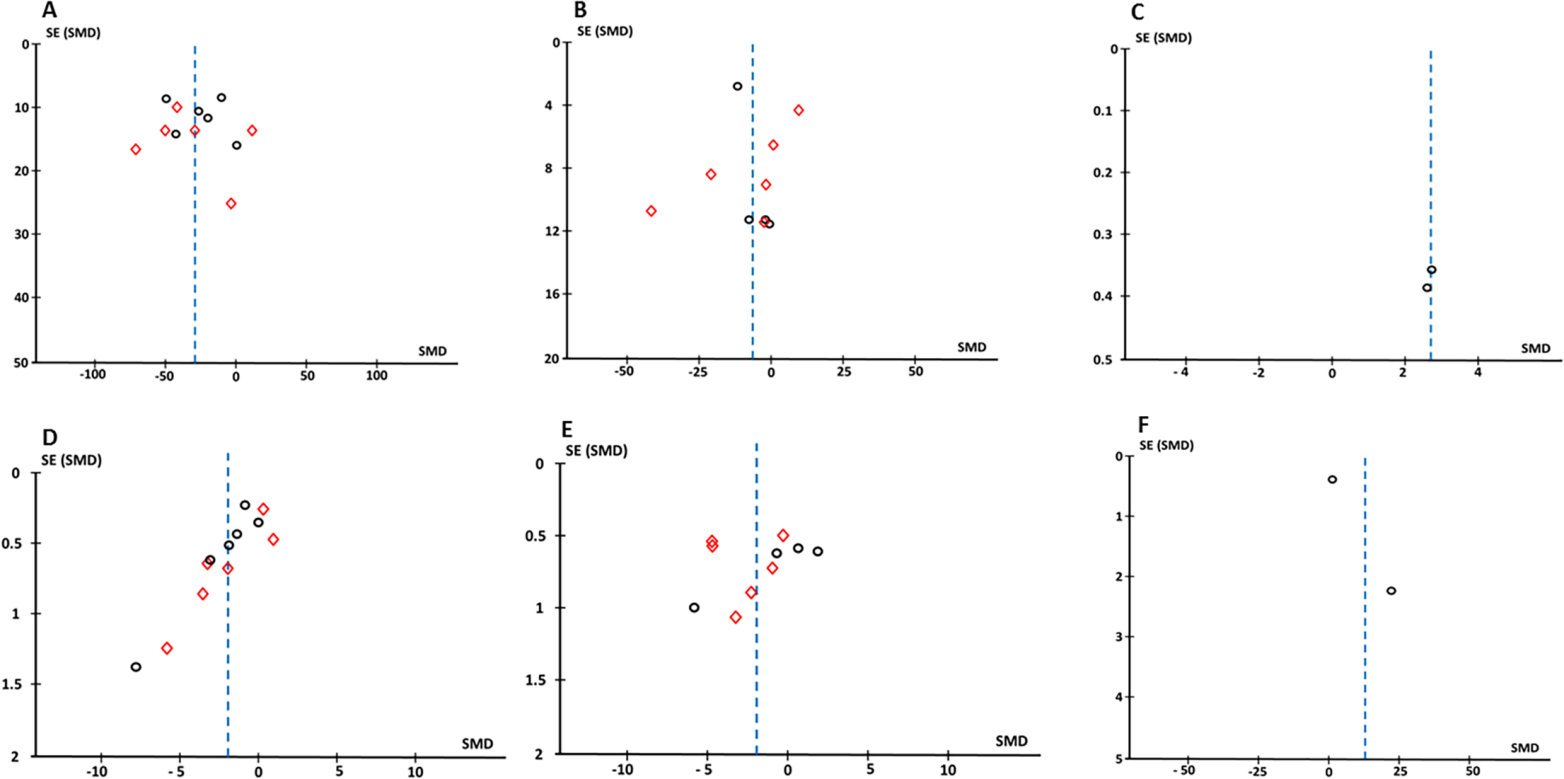
The funnel plots representative of publication bias. **After effects of c-tDCS on sensory threshold (A), pain threshold (B), and pain level (C) in patient group.** Also the publication bias in studies investigating sham effects of c-tDCS on sensory threshold (D), pain threshold (B), and pain level (E) in patient group are evaluated. In Figures A, B, D, and E, circles indicates S1 subgroup analysis and squares show M1 subgroup analyses.

Six funnel plots were generated to examine the result of each meta-analysis for evidence of publication bias. Three plots show the bias of included studies comparing after effects of c-tDCS on STh (Fig 8A) and PTh (Fig 8B) in healthy individuals, and PL (Fig 8C) in patients with chronic pain. As can be seen, the funnel plots are asymmetrical. In addition, funnel plots for the meta-analyses comparing the effect of active and sham c-tDCS on STh (Fig 8D), PTh (Fig 8E), and PL (Fig 8F) are also asymmetrical. These asymmetrical plots indicate the possibility of publishing studies with significant positive results and being reluctant to publish studies with non-significant results. Regarding the level of heterogeneity of meta-analyses in the current study, the other possibility is the small number of included studies and participants which often result in exaggerated or overestimated true effect size.

### Limitations of the study

The findings of current meta-analysis should be interpreted in the context of some limitations. First, the small sample sizes in some included studies were associated with larger effect sizes that might have affected the overall results and statistical significance. Second, Because of the scarcity of studies on the effect of c-tDCS on pain, it was impossible to analyses subgroups with fixed stimulation parameters. Finally, it is worth noting that our study considered only the immediate after effects of c-tDCS, not long-lasting effects. Due to the limited number of included studies and mismatched measurement time-points, it was impossible to evaluate long-lasting after effects of c-tDCS based on the site of stimulation.

### Areas for future research

The results of our study concern the immediate after effects of a single c-tDCS session. It is possible that longer applications or multiple applications could significantly increase PTh or decrease PL [69, 70]. Given the small number of clinical trials that have assessed the efficacy of c-tDCS to reduce pain level in patients with chronic pain, investigation of the effects of c-tDCS in patients with different pathologies would be useful. C-tDCS has short-term analgesic effects [59] so can be used in acute cases; as a result, opportunities exist for more studies of c-tDCS during its use to reduce acute and chronic pain in health centers.

## Supporting Information

S1 PRISMA Checklist PRISMA Checklist. (DOC)
